# Supplementary material for: A novel autotransfusion device saving erythrocytes and platelets used in a 72 h survival swine model of surgically induced controlled blood loss
Source: PLoS One. 2022 Mar 24;17(3):e0260855. doi: 10.1371/journal.pone.0260855 (PMC8947136; doi:10.1371/journal.pone.0260855)
Supplement: S1 File — (DOCX) [file pone.0260855.s001.docx]

**Supplemental materials**

**Appendix S1: Materials and Methods**

**Animal welfare**

This preclinical study was conducted in accordance with the regulations governing good laboratory practice: The Organisation for Economic Co-Operation and Development Principles of Good Laboratory Practice [1] and the United States Food and Drug Administration Good Laboratory Practice for Non-Clinical Laboratory Studies (FDA-CFR 21 Part 58) [2]. The animal study protocol was run according to the European Community Guidelines for the care and use of laboratory animals (2010/63/UE) [3] following approval by the Pays de La Loire ethical committee for animal experimentation and authorization by the French Ministry of Higher Education, Research and Innovation (Apafis number #11079-2017071317574824v1). All procedures were performed in an animal approved facility (Oniris Experimental Surgical Plateform, accreditation number C-44-271 by Loire Atlantique prefectoral order N° 2014-DDPP-132) and under guidance of the facility animal welfare body.

**Animals**

Adult female Yucatan mini-pigs (*Sus Scrofa domesticus)* weighing 40 to 55 kg were purchased from the French National Research Institute for Agriculture, Food and Environment breeding farm (INRAe Saint Gilles). Animals were housed in individual boxes and had a week of acclimatization before the experiment started. They were fed with an appropriate amount of specific food, according to the breeder recommendations (Pig Food Special miniature-pigs, INRAE Saint-Gilles). Water was delivered *ad libitum*. Mini-pig environment enrichment included a chewing disc, a metallic chain, a ball and abundant bedding with dust-free wood shavings. Handlers visited individual boxes three times a day to accustom the animals to their presence and perform positive reinforcement exercises to facilitate post-operative care. To limit animal stress due to pre-anesthetic fasting, feeding times were kept constant and induction of anesthesia was done just prior to morning feeding.

**Anesthesia, analgesia and animal instrumentation**

Animals were immobilized in their pen by an intramuscular injection of ketamine (10 mg/kg), midazolam (0.5 mg/kg) and methadone (0.5 mg/kg). Once unconscious, they were transported on a stretcher to the anesthesia preparation room where an oxygen facial mask (5 L/min) was applied and intravenous access gained by inserting a 22-gauge cannula in an auricular vein. Propofol (1-2 mg/kg to effect) was administered intravenously to allow orotracheal intubation (8 or 8.5 mm internal diameter cuffed tube). Anesthesia was maintained with a balanced technique: sevoflurane (ETsevo of 1.6-2.1 %) in an oxygen/air mixture (FiO_2_ 45 %) delivered in a circle rebreathing system (Cato^®^ Anaesthesia Workstation, Draeger France) and continuous infusions of lidocaine (3-6 mg/kg/h), ketamine (0.5-1 mg/kg/h) and midazolam (0.5-1 mg/kg/h). Methadone (0.1-0.25 mg/kg IV) was re-dosed to effect during the procedure. Propofol (0.1-0.2 mg/kg/min IV) was used in place of sevoflurane during bypass. Controlled ventilation was instituted to maintain normocapnia (EtCO_2_ of 35-40 mmHg) with a rate of 10-12 breaths per minute and a tidal volume of 10 mL/kg. Ventilatory parameters and gas flow during bypass were adjusted according to arterial blood gas measurement results. Monitoring with a multi-parameter monitor (Carescape^TM^ B650, GE Healthcare France) included temperature, ECG, pulse oximetry, respiratory gas measurement (CO_2_, O_2_, Sevoflurane), non-invasive blood pressure during animal instrumentation and invasive blood pressure during surgery. Depth of anesthesia was assessed using classical signs of myorelaxation like absence of palpebral reflex and lack of jaw tone as well as unresponsiveness to surgical stimulation and adjusted to maintain a stable plane of anesthesia.

A cut down to the right neck was performed to insert long term catheters (16G 15 cm Arrow^®^ Single lumen Central Venous Catheter) in the carotid artery and the jugular vein and left in place until the end of the experiment. The intra-arterial catheter allowed for invasive blood pressure monitoring and blood gas sampling. The jugular catheter was used to monitor central venous pressure, collect blood samples intra and post-operatively and administer the autotransfusion.

Intravenous lactated ringer solution was administered during anesthesia at a rate of 2 to 5 ml/kg/h. During the induced hemorrhage, mean arterial blood pressure was maintained at 60 mmHg by adjusting depth of anesthesia, dobutamine (0.5-4 µg/kg/min) and/or norepinephrine (0.05-0.2 µg/kg/min) administration tailored to effect and increased lactated ringer rate (10-20 mL/kg/h). If blood pressure at the end of the bleeding episode or after transfusion remained unstable, hypertonic saline 10 % (1 mL/kg IV) was added to maintain normotension.

Transfusion was administered by natural gravity following collected blood treatment by the autotransfusion device and was initiated once blood loss was stopped. The time, and duration of transfusion as well as animal hemodynamic constants were monitored during the infusion.

General anesthesia was stopped at the end of surgery or transfusion if it outlasted surgery completion. Animals were initially maintained in the operating room and monitored in sternal recumbency until return of spontaneous ventilation and brisk palpebral reflex. They were then transported to their recovery pen and kept under constant monitoring.

**Postoperative care for the abdominal model**

For the abdominal surgery, initial post-operative analgesia administered at the end of anesthesia included meloxicam (0.4 mg/kg IV), bupivacaine (2 mg/kg) infiltrated in the surgical wound at the end of surgery and buprenorphine (0.02 mg/kg IV). Meloxicam (0.4 mg/kg per os) was then administered once daily until euthanasia. Pain was assessed by evaluating overall animal behavior, response to human-animal interactions and careful palpation of the surgical zone every hour for the first 6 hours then four times a day until the end of the experiment. Rescue analgesia consisted of buprenorphine (0.02 mg/kg IV) and paracetamol (10 mg/kg orally).

**Postoperative care for the cardiac model**

For the cardiac procedure, post-operative analgesia starting at the end of surgery included meloxicam (0.4 mg/kg IV) once a day and buprenorphine (0.02 mg/kg IV) every 6 hours for the first 18 hours and paracetamol (10 mg/kg IV) twice to three times a day until euthanasia. A wound catheter (16G 15 cm diffusion catheter, Mila International Inc, USA) was placed between the muscle layers and the skin at the thoracotomy site and connected to an elastomeric pump (Easypump^®^ II, Bbraun, France) delivering 2 mL/h of lidocaine 2 % (40 mg/h). Pain was monitored continuously during intensive care then four times a day until the end of the experiment. Rescue analgesia consisted of supplemental administrations of buprenorphine (0.02 mg/kg IV) and paracetamol (10 mg/kg IV). Intravenous fluid therapy with lactated ringer solution supplemented with potassium chloride (20 meq/mL) at 3-4 mL/kg/h was maintained for the first 18 to 24 h until the animals were able to eat and drink on their own. Nasal oxygen supplementation (40-100 mL/kg/min) was titrated to effect depending on arterial blood gas results.

**Postoperative follow-up**

A postoperative follow-up of 72 ± 4 hours was carried out on each animal to detect any complication following the surgery and the treated blood reinfusion. A physical examination of the animal and blood sampling were performed between 2 h and 6 h postoperatively, between 6 h and 12 h postoperatively, and then every 12 hours until euthanasia at 72 h post-transfusion (7 blood sampling times). Different parameters were monitored on the mini-pigs: overall behavior and attitude, presence and intensity of pain, appetite and water consumption, body temperature, heart rate, respiratory rate, non-invasive blood pressure until animals were able to stand up, surgical wound aspect and presence of any hemorrhage. Any adverse effects were recorded and any given medication was noted.

**Euthanasia**

At the end of the 72 h post-transfusion time or earlier in case of reaching a humane endpoint, minipigs were re-anesthetized with an IV combination of ketamine (5 mg/kg), midazolam (0.5 mg/kg) and methadone (0.3 mg/kg) to be exsanguinated by transection of the abdominal aorta and the inferior vena cava. Massive hemorrhage leading to death under general anesthesia is an AVMA-approved method of euthanasia [4]. Depth of anesthesia was monitored during the process and adjusted by IV redosing of ketamine (5 mg/kg) and additional administration of propofol (0.05-0.1 mg/kg/min). Exsanguination improves macroscopic post-mortem organ evaluation, limits post-mortem clot formation in the vasculature and facilitates histologic examination especially when looking for signs of thrombosis [5].

**Post-mortem examination**

A systematic post-mortem examination was performed on each animal that documented

- presence of any effusion into the abdominal and thoracic cavities;

- gross aspect of the following organs observed in place and after their removal from the body: heart, liver, both kidneys, spleen (cardiac model only), lungs, at least two different thoracic lymphatic nodes. Any gross change observed in these listed organs was recorded. Tissue samples including large vessels into these organs were harvested from each one of them, allowing 5 to 10 sections to be performed per organ for further pathohistological analysis. If a tissue abnormality was observed, in particular evoking a potential thrombus, a tissue sample of the incriminated area was made and fixed in formaldehyde for a further histopathology analysis. The thrombogenic risk assessment associated with the use of the autotransfusion in this study was performed according to the ISO standard 10933-4 [6] and the application of the FDA guide: Use of International Standard ISO 10993-1, "Biological evaluation of medical devices - Part 1: Evaluation and testing within a risk management process", section thrombogenicity, published in June 2016 and updated in 2021 [7].

**References**

1. OECD Series on Principles of Good Laboratory Practice (GLP) and Compliance Monitoring - OECD. In: 1999 [Internet]. Available: <https://www.oecd.org/chemicalsafety/testing/oecdseriesonprinciplesofgoodlaboratorypracticeglpandcompliancemonitoring.htm>
2. United States Food and Drug Administration Good Laboratory Practice for Non-Clinical Laboratory Studies (FDA-CFR 21 Part 58). Available: <https://www.accessdata.fda.gov/scripts/cdrh/cfdocs/cfcfr/CFRSearch.cfm?CFRPart=58>
3. European Community Guidelines for the care and use of laboratory animals (2010/63/UE). 2010. doi: 10.1046/j.1537-2995.1996.36196190516.x
4. American Veterinary Medical Association Panel on Euthanasia. AVMA Guidelines for the euthanasia of animals: 2020 edition. 2020. Available: <https://www.avma.org/resources-tools/avma-policies/avma-guidelines-euthanasia-animals>
5. Renne RA, Everitt JI, Harkema JR, Plopper CG, Rosenbruch M, Pathology T, et al. OECD Guidance Document on Histopathology for Inhalation Studies Draft OECD Guidance Document on Histopathology for inhalation toxicity studies, Supporting TG 412 (Subacute Inhalation Toxicity: 28-Day) and TG 413 (Subchronic Inhalation Toxicity: 90-Day). 2009. Available: <https://www.oecd.org/chemicalsafety/testing/43822718.pdf>
6. ISO - ISO 10993-4:2017 - Biological evaluation of medical devices — Part 4: Selection of tests for interactions with blood. 2017. Available: <https://www.iso.org/standard/63448.html>
7. Use of International Standard ISO 10993-1, “Biological evaluation of medical devices-Part 1: Evaluation and testing within a risk management process” Guidance for Industry and Food and Drug Administration Staff Preface Public Comment. 2020. Available: <https://www.fda.gov/vaccines-blood-biologics/guidance-compliance-regulatory-information-biologics>

Table S1: Hematology results expressed as median (min-max) for the visceral and cardiac models (n=5 and n=4 respectively) at the different sampling times on the animals: during anesthesia before blood loss (T0) and after autologous re-transfusion (TP), then during the 72 h survival follow-up period (7 sampling times following the end of the transfusion).

| **Time /**  **Parameters** | **Surgical Models** | **T0** | **TP** | **T2-6h** | **T6-12h** | **T12-24h** | **T24-36h** | **T36-48h** | **T48-60h** | **T60-72h** |
| --- | --- | --- | --- | --- | --- | --- | --- | --- | --- | --- |
| **Red bloods cells (10^6^/**𝝁**L)** | VISC | **4.6**  *(4.3-6.1)* | **5.4**  *(5.3-5.8)* | **5.6**  *(5.3-5.9)* | **5.5**  **(5.1-5.7)** | **5.7**  *(5.1-6.2)* | **5.7**  *(5-6)* | **5.3**  **(***4.9-5.4)* | **5.3**  *(4.7-5.7)* | **5.3**  *(5-5.9)* |
|  | CARD | **4.6**  *(4.3-5.1)* | **4.3**  *(4.1-4.5)* | **4.6**  *(3.9-4.8)* | **3.9**  *(3.4-4.5)* | **4.4**  *(3.6-4.8)* | **3.9**  *(3.4-4.5)* | **4.5**  *(4.1-4.9)* | **4.1**  *(3.3-5.2)* | **3.9**  *(3.5-4.2)* |
| **Hemoglobin**  **(g/L)** | VISC | **93**  *(85-119)* | **111**  *(105-114)* | **112**  *(104-124)* | **112**  *(105-116)* | **117**  *(103-121)* | **112**  *(101-119)* | **106**  *(98-110)* | **105**  *(94-112)* | **105**  *(98-113)* |
|  | CARD | **94.5**  *(90-105)* | **88.5**  *(85-93)* | **94**  *(81-97)* | **80**  *(73-90)* | **92**  *(75-98)* | **79**  *(71-91)* | **93.5**  *(85-98)* | **83.5**  *(66-109)* | **79**  *(73-87)* |
| **Hematocrit  (%)** | VISC | **29**  *(27-39)* | **35**  *(34-38)* | **37**  *(34-40)* | **36**  *(33-37)* | **37**  *(33-41)* | **35**  *(32-38)* | **33**  *(31-36)* | **34**  *(30-36)* | **33**  *(32-37)* |
|  | CARD | **30**  **(29-33)** | **28**  *(26-30)* | **30**  *(26-31)* | **25**  *(23-29)* | **28.5**  *(23-30)* | **24.5**  *(22-28)* | **30**  *(27-32)* | **27**  *(21-35)* | **26**  **(***24-29)* |
| **VGM**  **(fL)** | VISC | **63.8**  *(62.2-66.7)* | **64.8**  *(63.8-67.8)* | **65.9**  *(64.5-68.4)* | **65**  *(64.2-66.8)* | **65.4**  *(62.6-65.8)* | **64.4**  *(61.8-66)* | **64.1**  *(62.2-66.9)* | **63.7**  *(61.6-66.9)* | **64.1**  *(62-70.1)* |
|  | CARD | **65.85**  *(64.4-66.6)* | **64.4**  *(63.9-66.3)* | **65.8**  *(63.5-66.7)* | **65.4**  *(63.1-66.1)* | **64.5**  *(62.8-66)* | **64.1**  *(62.1-65.7)* | **66.45**  *(64.3-68.1)* | **66.2**  *(62.8-68.2)* | **67.2**  *(67-70.5)* |
| **TCM**  **(pg)** | VISC | **20**  *(19.4-20.8)* | **20.2**  *(19.5-21.1)* | **20.2**  *(19.9-21.2)* | **20.5**  *(19.9-21.2)* | **20.1**  *(19.6-21)* | **20.2**  *(19.5-20.9)* | **20.1**  *(19.6-20.7)* | **19.9**  *(19.4-20.7)* | **19.8**  *(19.3-21.1)* |
|  | CARD | **20.7**  *(20.2-21)* | **20.7**  *(19.8-21.3)* | **20.5**  *(20-21.1)* | **20.7**  *(20.1-21.7)* | **20.7**  *(20.4-21.2)* | **20.7**  *(19.9-21.3)* | **20.7**  *(20.2-21.2)* | **20.6**  *(19.8-21.1)* | **20.6**  *(20.2-21)* |
| **CCMH**  **(%)** | VISC | **31.6**  *(30.4-32.2)* | **31.1**  *(30.2-31.6)* | **31**  *(30.2-31.5)* | **31.5**  *(30.6-32)* | **31.6**  *(29.9-32.1)* | **31.4**  *(30.2-32)* | **31.4**  *(30.9-31.8)* | **31.3**  *(30.9-32)* | **30.8**  *(30-31.7)* |
|  | CARD | **31.4**  *(31.3-31.7)* | **31.7**  *(31-33)* | **31.3**  *(30.9-31.9)* | **32.1**  *(30.9-32.8)* | **32.3**  *(31.7-32.7* | **32.2**  *(32-32.6)* | **31.1**  *(30.8-32)* | **31**  *(31-31.6)* | **30.2**  *(29.8-30.9)* |
| **Reticulocytes**  **(10^3^/𝝁L)** | VISC | **62.3**  *(44.8-114.1)* | **102.2**  *(91.7-144.2)* | **120.5**  *(96.5-147.4)* | **114**  *(1007-144.8)* | **180.8**  *(141.4-213.4)* | **199.1**  *(161.3-233.3)* | **213.6**  *(201.1-236.2)* | **222.8**  *(186-244)* | **191**  *(116.9-238.1)* |
|  | CARD | **42.45**  *(28.9-60.8)* | **25.8**  *(19.6-40.5)* | **69.3**  *(57.8-86.5)* | **59.9**  *(39.2-90.8)* | **68.4**  *(34.6-116.3)* | **55.2**  *(32.4-90.2)* | **94.8**  *(67.9-121.7)* | **100.5**  *(73.5-165.1)* | **75.3**  *(66-84.5)* |
| **White Blood cells**  **(10^3^/𝝁L)** | VISC | **10.7**  *(6.6-18.6)* | **9.5**  *(5.3-12.2)* | **22.7**  *(15.8-27.9)* | **28.2**  *(19.6-28.7)* | **22.4**  *(18.9-26.5)* | **24.8**  *(19.6-25.2)* | **16.2**  *(15.4-16.9)* | **16.9**  *(15.3-17.4)* | *15.2*  *(14.7-21.1)* |
|  | CARD | **11.9**  *(6.8-18.4)* | **14.1**  *(12.2-20.1)* | **21.5**  *(16-29.5)* | **26.3**  *(16.8-35)* | **22.6**  *(16.1-28.1)* | **22.7**  *(13-26.2)* | **17.5**  *(15.2-20.9)* | **18.8**  *(17.5-22.4)* | **17.4**  *(12.7-23.3)* |
| **Neutrophils**  **(10^3^/𝝁L)** | VISC | **5.3**  *(3.2-11.6)* | **5.1**  *(3.2-7.6)* | **16.7**  *(12.3-25)* | **24.7**  *(17.2-26.4)* | **17.9**  *(12.9-21.8)* | **18.6**  *(11.7-20)* | **9.5**  *(9.4-10.9)* | **9.8**  *(7.9-11.5)* | **8.5**  *(6.7-11.4)* |
|  | CARD | **6.8**  *(2.2-11.1)* | **10.7**  *(7.9-13.5)* | **17.1**  *(12.4-25.6)* | **22.6**  *(16.3-32.8)* | **18.4**  *(12.3-23.2)* | **15.7**  *(8.9-18.3)* | **10.8**  *(9-12.3)* | **10.3**  *(9.7-14.1)* | **11.0**  *(6-18.6)* |
| **Lymphocytes**  **(10^3^/𝝁L)** | VISC | **4.3**  *(2-6.2)* | **3.1**  *(1.8-4.1)* | **3**  *(0.9-5.1)* | **1.4**  *(0.8-2.9)* | **3.3**  *(2.9-4.2)* | **4.1**  *(3.3-4.7)* | **3.8**  *(3.5-4.6)* | **4.5**  *(3.8-5.1)* | **5.2**  *(4.2-6.4)* |
|  | CARD | **4.3**  *(3.8-5.8)* | **3.5**  *(3-5.7)* | **3.1**  *(2.8-4.6)* | **1.5**  *(0.3-3.8)* | **3.3**  *(2.8-3.6)* | **4.6**  *(3.2-5.7)* | **4.7**  *(3.9-5.4)* | **5.7**  *(4.6-7.1)* | **3.9**  *(2.7-5.4)* |
| **Monocytes**  **(10^3^/𝝁L)** | VISC | **0.6**  *(0.2-1.1)* | **0.4**  *(0.3-1)* | **0.7**  *(0.5-2)* | **0.9**  *(0.6-1.3)* | **1.1**  *(0.7-1.7)* | **1.3**  *(1-2.1)* | **1.4**  *(1-1.8)* | **1.9**  *(1.4-2.5)* | *1.8*  *(1-2.5)* |
|  | CARD | **0.7**  *(0.6-0.9)* | *0.5*  *(0.3-0.8)* | **0.7**  *(0.5-0.8)* | **0.8**  *(0.2-1.2)* | **1.0**  *(0.5-1.1)* | **1.3**  *(0.5-1.4)* | **1.5**  *(1.2-1.9)* | *1.6*  *(0.5-2.1)* | **1.3**  *(1.1-1.6* |
| **Eosinophils**  **(10^3^/𝝁L)** | VISC | **0.1**  *(0-0.2)* | **0**  *(0-0.1)* | **0**  *(0-0.2)* | **0**  *(0-0)* | **0.1**  *(0-0.1)* | **0.4**  *(0.2-1.1)* | **0.9**  *(0.5-1.3)* | **0.7**  *(0.5-0.9)* | **0.7**  *(0.7-0.9)* |
|  | CARD | **0.3**  *(0-0.6)* | **0**  *(0-0.1)* | **0**  *(0-0.1)* | **0**  *(0-0)* | **0.1**  *(0.1-0.2)* | **0.8**  *(0.4-1.6)* | **1**  *(0.2-1.3)* | **1**  *(0.7-1.6)* | **0.8**  *(0.2-1.3)* |
| **Basophils**  **(10^3^/𝝁L)** | VISC | **0**  *(0-0.1)* | **0**  *(0-0)* | **0**  *(0-0)* | **0**  *(0-0)* | **0**  *(0-0)* | **0**  *(0-0)* | **0**  *(0-0)* | **0**  *(0-0)* | **0**  *(0-0)* |
|  | CARD | **0**  *(0-0)* | **0**  *(0-0)* | **0**  *(0-0)* | **0**  *(0-0)* | **0**  *(0-0)* | **0**  *(0-0)* | **0**  *(0-0)* | **0**  *(0-0)* | **0**  *(0-0)* |
| **Platelets**  **(10^3^/𝝁L)** | VISC | **331**  *(295-383)* | **246.5**  *(224-306)* | **231.5**  *(163-269)* | **210**  *(156-262)* | **244.5**  *(196-295)* | **233**  *(200-244)* | **238**  *(207-318)* | **241**  *(198-319)* | **281.5**  *(256-294)* |
|  | CARD | **336**  *(287-351)* | **289.5**  *(241-304)* | **275**  *(265-297)* | **222.5**  *(198-279)* | **266**  *(196-293)* | **292.5**  *(252-314)* | **294.5**  *(262-382)* | **325.5**  *(288-367)* | **316**  *(280-383)* |

Table S2: ROTEM-INTEM results expressed as median (min-max) for the visceral and cardiac models (n=5 and n=4 respectively) at the different sampling times on the animals: during anesthesia before blood loss (T0) and after autologous re-transfusion (TP), then during the 72 h survival follow-up period (7 sampling times following the end of the transfusion).

| **Parameters/Time** | | **Surgical Models** | **T0** | **TP** | **T2-6h** | **T6-12h** | **T12-24h** | **T24-36h** | **T36-48h** | **T48-60h** | **T60-72h** |
| --- | --- | --- | --- | --- | --- | --- | --- | --- | --- | --- | --- |
| **INTEM** | **CT**  **(s)** | VISC | **153**  *(125-160)* | **183**  *(151-275)* | **132**  *(99-145)* | **122**  *(95-214)* | **136**  *(121-185)* | **199**  *(132-225)* | **199**  *(170-308)* | **215**  *(150-230)* | **216**  *(163-319)* |
|  |  | CARD | **153**  *(109-164)* | **186**  *(133-215)* | **177**  *(144-194)* | **154**  *(136-185)* | **133**  *(105-179)* | **172**  *(137-213)* | **199**  *(144-214)* | **179**  *(150-272)* | **251.5**  *(204-334)* |
|  | **A30**  **(mm)** | VISC | **73**  *(69-74)* | **63**  *(57-65)* | **63**  *(58-65)* | **64**  *(60-66)* | **65**  *(64-68)* | **68**  *(65-71)* | **67**  *(65-73)* | **69**  *(68-72)* | **71**  *(66-74)* |
|  |  | CARD | **70.5**  *(64-77)* | **69**  *(64-71)* | **66.5**  *(64-69)* | **66.5**  *(66-68)* | **69.5**  *(69-73)* | **72.5**  *(70-73)* | **72.5**  *(60-75)* | **73.5**  *(72-77)* | **75**  *(74-77)* |
|  | **MCF**  **(mm)** | VISC | **74**  *(72-77)* | **65**  *(59-71)* | **67**  *(61-68)* | **67**  *(63-71)* | **71**  *(69-73)* | **74**  *(72-76)* | **74**  *(71-80)* | **75**  *(73-77)* | **77**  *(72-78)* |
|  |  | CARD | **73.5**  *(68-79)* | **71**  *(66-73)* | **70**  *(67-72)* | **71**  *(70-72)* | **74.5**  *(73-77)* | **76**  *(75-77)* | **76**  *(64-79)* | **77.5**  *(75-80)* | **79**  *(78-80)* |
|  | **Alpha Angle**  **(°)** | VISC | **81**  *(81-82)* | **77**  *(75-80)* | **78**  *(76-80)* | **77**  *(76-80)* | **78**  *(75-80)* | **78**  *(77-79)* | **78**  *(75-82)* | **79**  *(76-81)* | **80**  *(76-81)* |
|  |  | CARD | **80.5**  *(79-83)* | **79**  *(78-81)* | **79**  *(77-80)* | **78.5**  *(76-79)* | **78.5**  *(76-80)* | **77.5**  *(76-79)* | **76.5**  *(74-79)* | **77**  *(75-81)* | **78.5**  *(78-79)* |
