# Supplementary material for: A novel autotransfusion device saving erythrocytes and platelets used in a 72 h survival swine model of surgically induced controlled blood loss
Source: PLoS One. 2022 Mar 24;17(3):e0260855. doi: 10.1371/journal.pone.0260855 (PMC8947136; doi:10.1371/journal.pone.0260855)
Supplement: Attachment — Submitted filename: Response to Reviewer.docx [file pone.0260855.s003.docx]

PONE-D-21-23852R1

A novel autotransfusion device saving erythrocytes and platelets by filtration used in a 72 h survival swine model of controlled blood loss: perioperative hematologic and coagulation assessments, salvaged blood characteristics and system performance

PLOS ONE

Response to Reviewers – Revision 2

Reviewer #3: I believe the paper was significantly improved with the revision.
There are however some points that need to be addressed, as follows.

**Major points**
- The title is too long. Is it possible to shorten?

The title has been shortened and is now as follow :

“A novel autotransfusion device saving erythrocytes and platelets used in a 72h survival swine model of surgically induced controlled blood loss”

- The Materials and Methods section is too long, and very exhaustive to read. It would benefit of shortening to about a half or 1/3, eventually by moving part to the supplemental material

The Material and Methods section has been shortened. The details on the anesthesia and postoperative management have been transferred in the supplemental data section.

- Compared to the visceral model, the platelet concentration was higher in the cardiac model, but as expected, the platelet yield was lower, which can be attributed to the deleterious effect of CBP on platelets, as discussed by the authors.

Thank you for the acknowledgement

- The authors mention that there was no platelet activation during the treatment, and platelet kept the ability to be activated by treatment, confirmed in their previous study with human blood. Did the authors confirm this finding with the pig platelets? If yes, the results should be described, and if not, it should be mentioned in the Discussion.

We could not test platelet activation in the transfusion bag after the treatment because we had no access to specific platelet activation evaluation test. Indeed, plasma protein are washed and eliminated in the treated blood. Then fibrinogen was eliminated and aggregation tests with the ROTEM platelets were not suitable. Flow cytometry could have been used as in the previous study on human blood. However, no flow cytometer was available on the experimentation site. The following sentence was added to the manuscript:

“The reinfusion of platelets is considered as a potential prothrombotic risk. However, it was shown that the i-SEP ATS does not activate platelets during the treatment, and that platelets keep their ability to be activated after the treatment [30]. However, platelet activation was not evaluated in this animal study.”

- In Page 30, Line 684, there is mention to the hemolysis rate higher than the 0.8% threshold. However, there is no mention to the expected hemolysis rate in the Materials and Methods section. It should be described in Page 17, together with the other expected device performance indexes.

The 0.8% refers to the maximal expected hemolysis in packed red cells defined by the European guidelines (*European Directorate for the Quality of Medicines and HealthCare of the Council of Europe (EDQM): Guide to the Preparation, Use and Quality Assurance of Blood Components, 20th edition. Strasbourg, France, Council of Europe Publishing, 2020*). The reference of the guidelines and the references are added to the manuscript as follow :

“Despite the hemolysis rate higher than the 0.8% threshold (as defined by the European guidelines, reference added) and the RBCs yield below 80 %, the treated blood reached the 45 % to 65 % hematocrit reference range and its reinfusion was well tolerated by the animals.”

**Minor points**

In Page 14, Lines 326-328: [“Use of International Standard ISO 10993-1”, “Biological evaluation of medical devices – Part 1: Evaluation and testing within a risk management process”] should be [Use of International Standard ISO 10993-1, “Biological evaluation of medical devices – Part 1: Evaluation and testing within a risk management process”]
In Page 19, Line 450: (Table S1) should be (Tables S1 and S2, respectively)
In Page 25, Lines 586 and 587: [61.7 % (50.1-71.9)] should be [61.7 (50.1-71.9) %] and [70.5 % (65.4-72.1)] should be [70.5 (65.4-72.1) %]
In Table 4: the hemoglobin concentration should be 80 and 61 (g/L) and not 8.0 and 6.1
In Page 27, Line 612: [complication or nor] should be [complication nor]
In Page 28, Line 625: [ovine RBCs cells] should be [ovine RBCs]
In Page 37, Line 857: is [Internet] necessary? Please check
In Page 37, Line 868: the link is incomplete. Please check
In Page 39, Line 902: the link is incomplete. Please check
In Page 39, Line 904: is [Internet] necessary? Please check

All the minor points have been addressed.

Concerning internet links, after a new careful reading of the guidelines, our interpretation is that the journal prefers having the full link. However, it is a misreading on our part, we have identified the section to be remove in yellow in the document “revised manuscript”

Thank you for your time and very thoughtful suggestions
